# Potential economy-wide impacts of an African swine fever outbreak in the United States

**DOI:** 10.3389/fvets.2026.1752899

**Published:** 2026-02-02

**Authors:** Tais C. de Menezes, Amanda M. Countryman, Dustin L. Pendell, Jonathan Rushton, Jimmy Tickel, Heather Simmons

**Affiliations:** 1 Department of Agricultural and Resource Economics, Colorado State University, Fort Collins, CO, United States; 2 Department of Agricultural Economics, Kansas State University, Manhattan, KS, United States; 3 The Global Burden of Animal Diseases Programme, The Roslin Institute, The University of Edinburgh, Midlothian, United Kingdom; 4 Institute for Infectious Animal Diseases, Texas A&M University, College Station, TX, United States

**Keywords:** African swine fever, agricultural trade disruptions, computable general equilibrium model, livestock disease impacts, U.S. pork industry

## Abstract

**Introduction:**

African swine fever (ASF) poses a major threat to the U.S. pork sector, with potentially large spillovers through domestic markets and international trade. To inform preparedness and policy design, we quantify the economy-wide consequences of a hypothetical ASF outbreak under alternative scenarios of production losses and export restrictions.

**Methods:**

We use a computable general equilibrium (CGE) model to simulate four hypothetical ASF scenarios that vary by outbreak size and export restrictions. The model endogenously captures adjustments in production, prices, bilateral trade flows, and welfare, and we assess robustness using systematic sensitivity analysis.

**Results:**

Small outbreak scenarios generate limited sectoral disruption and no substantial GDP effects, with U.S. welfare losses of $310–$563 million. Large outbreak scenarios reduce U.S. hog production by 7.34%–8.57% and pork production by 7.25%–10.8%, increase U.S. hog producer prices by 41.6%–41.8% and pork prices by 6.5%, and reduce U.S. GDP by 0.05%. Corresponding U.S. welfare losses rise to $10.9–$11.4 billion. Globally, large outbreaks increase world hog and pork prices by 3.67%–3.69% and 1.20%–1.22%, respectively, and drive trade diversion toward alternative exporters.

**Discussion:**

The results indicate that the economic costs of ASF increase substantially with outbreak size and are shaped by the severity of trade restrictions. Investments that prevent escalation (early detection, rapid containment) and strategies that preserve market access (credible regionalization/zoning arrangements) can substantially reduce welfare losses and global market disruption.

## 1 Introduction

African swine fever (ASF) is a highly infectious viral hemorrhagic disease that affects pigs and wild boars, resulting in relatively high mortality rates. The virus transmission occurs through direct animal contact, contaminated fomites, and biological vectors (1). ASF outbreaks trigger immediate biosecurity measures, as mandated by international animal health protocols, and trade restrictions (2, 3). These disruptions cascade through global agricultural markets, causing substantial economic losses for hog producers, related industries, and trade partners. The disease’s persistence in the environment and lack of effective vaccines make containment particularly challenging, requiring aggressive control measures that further impact agricultural trade and food security (4–6).

The global spread of ASF has heightened concerns among major pork-exporting nations regarding its potential impact on international trade and market stability. The 2018–2019 ASF outbreak in China, which led to the culling of 1.2 million hogs and economic losses exceeding $200 billion, highlights the devastating consequences of the disease (7). Following China’s outbreak, ASF expanded through Southeast Asia and subsequently emerged in European territories. After a four-decade absence in the Americas, ASF reappeared in 2021 with confirmed cases in the Dominican Republic and Haiti (4, 8). This reemergence in the Caribbean region has raised significant biosecurity concerns among neighboring countries, particularly given the disease’s potential for rapid transboundary spread and its severe economic implications for the Americas’ substantial pork industry. The proximity of these outbreaks to major pork-producing regions, such as the United States (U.S.), Canada, and Brazil, has intensified surveillance efforts and biosecurity protocols throughout the Americas (5, 9, 10).

In response to the ASF outbreak in the Dominican Republic, the U.S. Department of Agriculture (USDA) established a protection zone in Puerto Rico and the U.S. Virgin Islands, suspending all movements of swine and swine products from these territories to the U.S. mainland (11). The U.S. has maintained heightened ASF vigilance due to its potential negative economic implications at both territorial and mainland levels. An ASF outbreak in Puerto Rico or the U.S. Virgin Islands would trigger international trade restrictions affecting the entire nation, regardless of mainland disease status. As the world’s third-largest pork producer after China and the European Union, and second-largest pork exporter, the U.S. faces substantial economic risk from potential ASF introduction. The disease could severely disrupt domestic production capacity and compromise the nation’s competitive position in global pork markets, resulting in long-term loss of market share and extensive economic damage to the U.S. agricultural sector (12–15).

Although existing studies explore ASF’s economic impacts, significant gaps remain. For example, Carriquiry et al. (12) examine the impacts of a hypothetical ASF outbreak in the U.S. using partial equilibrium and input–output models, highlighting severe long-term economic disruptions under prolonged export bans, without accounting for potential changes in bilateral trade. Earlier work by Rendleman and Spinelli (16) demonstrated the economic benefits of ASF prevention programs in the U.S., but their findings may not apply to recent events due to changes in U.S. pork production and global markets in the past two decades. Furthermore, recent studies for the U.S. by Lee et al. (17) and Kashyap, Suter, and McKee (18) focus primarily on consumer impacts, not accounting for broader economy-wide effects.

There is also a notable gap in the literature regarding the use of computable general equilibrium (CGE) models to evaluate the economic impacts of ASF in the U.S. Existing CGE-based analyses of ASF focus almost exclusively on outbreaks in China. For instance, Ahmed (19) examines the economic impacts of ASF in China and its ripple effects on global agricultural markets, using the GTAP-AGR CGE model. Similarly, Mason-D’Croz et al. (20) explore the global economic consequences of a major ASF outbreak in China, combining the IMPACT partial equilibrium model and the GLOBE CGE model and offer insights into the resilience and vulnerabilities of the global food system during severe production shocks. While these studies provide important insights into international transmission channels and global adjustment mechanisms, their findings are not applicable to the U.S. To our knowledge, no existing study has employed CGE models to assess the economic impacts of a hypothetical ASF outbreak in the U.S., despite the country’s central role in global pork markets and the potentially large macroeconomic and welfare consequence of such an event.

This study examines the economic implications of a hypothetical ASF outbreak in the U.S., focusing on the effects on domestic and global pork markets. Using a CGE framework, we assess potential changes in production output, prices, trade flows, and welfare. By quantifying these economy-wide impacts, this research provides critical insights for policymakers and stakeholders to inform disease prevention and response strategies. Our study addresses research gaps by leveraging the Global Trade Analysis Project (GTAP) CGE model to provide a robust framework for analyzing ASF’s potential economic impacts on domestic and global markets. By integrating production and bilateral trade data, the research not only investigates ASF-specific challenges but also contributes a robust tool for evaluating the economic consequences of other highly infectious animal diseases.

## 2 Materials and methods

The GTAP model is a multi-region, multi-sector CGE model that captures complex economic relationships across different sectors and regions of the global economy (21). The GTAP modeling framework represents the world economy through detailed national-level economic data, bilateral trade flows, and a multitude of behavioral parameters that determine how different agents (households, firms, and governments) respond to changes in prices and market conditions. The GTAP model incorporates key economic behaviors including consumer demand patterns, firm production decisions, and international trade relationships, while ensuring market clearing conditions and macroeconomic consistency (22). This framework connects countries and regions via trade relationships, capturing the cross-border effects of policy changes, production shocks, or market disruptions.

While partial equilibrium (PE) models focus on specific markets or sectors, CGE models such as GTAP provide a broader, economy-wide perspective and intersectoral linkages. For instance, when analyzing agricultural shocks, GTAP can capture not only the direct effects on producers and meat processors but also the indirect impacts on other agricultural sectors and even seemingly unrelated industries that might be affected through factor market competition or income effects. In addition, the GTAP model incorporates bilateral trade flows, which allows for the explicit modeling of trade linkages between regions. This feature captures the interplay of regional supply and demand structures with international trade dynamics, enabling the analysis of how changes in one region’s policies or shocks affect global trade patterns and other regions’ economies. By including these bilateral trade relationships, this framework provides a broad view of the global economy, accounting for substitution effects and trade diversion (23).

The GTAP model is particularly well-suited for evaluating the economic impacts of ASF in the U.S. for several reasons. First, the model’s detailed representation of agricultural sectors and livestock supply chains allows it to capture production losses associated with an ASF outbreak. Second, GTAP’s global trade linkages can accurately represent how trade restrictions and market access changes following an ASF outbreak would affect both U.S. exports and the reallocation of global pork trade flows. Third, the model’s general equilibrium structure allows for important adjustment mechanisms such as macroeconomic implications for economies heavily dependent on pork production, including welfare measured by equivalent variation. Equivalent variation provides a way to measure how much money agents in an economy would need to maintain their original level of satisfaction (or welfare) after a change. These features make GTAP an important tool for providing policymakers with a comprehensive assessment of the potential economic consequences of an ASF outbreak, while helping identify vulnerable sectors and regions that might require targeted support measures (23).

The GTAP framework is a widely used tool for evaluating the economic impacts of animal diseases on global livestock and meat markets. Boisvert et al. (24) applied GTAP to analyze the economic repercussions of a hypothetical foot-and-mouth disease (FMD) outbreak in the U.S., highlighting its effects on production, trade, and regional economies. Similarly, Countryman and Hagerman (25) and de Menezes et al. (26) employed the framework to assess the economic impacts of FMD in Latin America and Brazil, respectively, emphasizing trade restrictions and welfare changes. De Menezes et al. (27) extended this work to explore the consequences of bovine spongiform encephalopathy (BSE) in Brazil, using GTAP to capture trade and welfare effects. Additionally, Countryman et al. (28) applied the GTAP model to evaluate the economy-wide effects of livestock disease mitigation in Ethiopia, demonstrating the framework’s versatility in capturing both direct and indirect impacts across sectors. These studies underscore the GTAP model’s capability to provide macroeconomic insights into the economic effects of livestock diseases, making it a valuable approach for analyzing the potential effects of ASF in the U.S.

Version 11 of the GTAP database includes 160 countries and regions and 65 sectors, using 2017 as the reference year (29). Due to the extensive nature of the dataset, the reference year lags behind the most recent years, as the collection and reconciliation of data inputs required to build the database demand substantial time and resources. We update the database to 2022 with macroeconomic projections for population and gross domestic product (GDP), consistent with standard practices in the literature (30, 31). Additionally, we disaggregate the “other animals” sector into “hogs” and “other animals” and split the “other animal products” sector into “pork” and “other animal products.” These modifications result in a database with 67 sectors, allowing for more targeted analysis of the pork industry for this analysis.

To enhance computational efficiency and address the practical limitations of analyzing 160 individual countries and regions, we aggregate them into 11 broader regions focusing on the key players in the global pork market as follows: the U.S.; Canada; Mexico; Brazil; China and Hong Kong; Rest of Asia and Oceania; South and Southeast Asia; Central and South America; Europe; Middle East and North Africa; and Central and Southern Africa. Similarly, we condense the 67 GTAP database sectors into nine aggregated categories: hogs, pork products, feed, other livestock and meat, other crops, processed food, mining and extraction, manufacturing, and services. This aggregation balances computational feasibility with the level of detail necessary to capture the key economic interactions and trade flows related to the impacts of ASF.

In summary, the current study’s approach underscores the flexibility and robustness of the GTAP model as a tool for economic assessment of animal disease effects. Our analysis offers nuanced insights into the global pork market and welfare by accounting for the distinctive roles and interactions of key market participants while evaluating the impacts of supply shifts and trade reallocations. By situating the analysis within the broader context of international trade, this research emphasizes the interconnected nature of global markets and the importance of coordinated responses to animal disease-related disruptions.

### 2.1 U.S. ASF outbreak and trade policy scenarios

Two hypothetical ASF outbreak scenarios are examined, a small outbreak and a large outbreak. Each outbreak scenario is combined with two corresponding trade policy restrictions that limit U.S. exports, resulting in four scenarios for economic evaluation. The four scenarios assess potential ASF-related production shocks resulting from swine death and culling and export restrictions due to ASF.

First, we consider the potential impacts of a small outbreak on production, corresponding to early detection and rapid containment, in which infection is identified quickly and control measures prevent widespread transmission beyond a localized area. In this scenario, we impose a 0.044% decrease in U.S. hog production, intended to reflect the potential impact of localized biosecurity measures or minor disruptions triggered by ASF. Although modest in percentage terms, a 0.044% reduction in the U.S. hog herd corresponds to approximately 33,100 animals, based on the 2023 national inventory. This magnitude is consistent epidemiological simulation evidence for early-stage ASF events in the U.S. For example, Sykes et al. (32) simulate ASF incursions under alternative surveillance and control regimes using data from three major firms that produce hogs in the U.S., and report median numbers of eliminated animals ranging from 8,800 to 27,177 head across four containment scenarios. Only in their most stringent control scenario does the median number of eliminated animals exceed 33,000 head (50,208 animals).

Literature on ASF outbreaks emphasizes that even low-intensity outbreaks or heightened surveillance protocols can result in marginal production declines due to precautionary culling, movement restrictions, or temporary inefficiencies in production processes (1, 2, 5). For instance, Juszkiewicz et al. (1) and USDA (5) highlight how localized ASF prevention zones can require precautionary measures such as targeted culling or the suspension of swine movements, which, while effective in disease containment, may marginally affect overall production levels. Similarly, studies of livestock disease impacts, such as Frezal, Gay, and Nenert (33) and Mason-D’Croz et al. (20), provide evidence that even small-scale disruptions can lead to measurable but minor adjustments in production.

This small production decrease also mirrors findings from broader studies on livestock disease mitigation. For example, Carriquiry et al. (12) demonstrate that heightened ASF-related precautions, even without widespread outbreaks, can cause minor production adjustments due to increased biosecurity protocols and market sensitivity. Additionally, studies on FMD (24, 25) underscore how localized containment measures can trigger slight yet significant declines in production The small production shock used here therefore lies within the range of early-detection containment outcomes reported in the literature, and reflects a plausible situation in which rapid response successfully limits disease spread but nonetheless generates nontrivial economic disruption. Importantly, this scenario is not intended to represent a widespread epidemic, but rather a localized ASF event with effective containment, serving as a lower-bound benchmark.

In the small ASF outbreak scenario, we consider two levels of trade restrictions: a 20% decrease (SO1) and a 40% decrease (SO2) in U.S. hog and pork exports (Table 1). This scenario is based on ASF’s classification as a notifiable disease under WOAH. As a result, the U.S. faces a significant risk of reduced exports due to two primary factors: adherence to WOAH’s guidelines, which often require temporary trade suspensions, and the likely imposition of unilateral trade restrictions by importing nations seeking to protect their domestic pork industries from potential disease risks. Historical evidence highlights the likelihood of swift and severe trade restrictions following ASF detection. For instance, in 2023, Armenia, Australia, Japan, the Philippines, Taiwan, and Ukraine completely halted pork imports from Sweden after ASF was detected in wild boars (34–36). These actions reflect the precautionary measures taken by importing countries, even when outbreaks are localized and confined to non-commercial populations. Such responses are not uncommon, as the fear of ASF spreading through imported pork products often prompts trading partners to impose embargoes, regardless of the exporting country’s ability to regionalize or mitigate the outbreak (27, 37, 38).

**Table 1:** Shocks to U.S. hog and pork production and exports.

Sector	Small ASF outbreak		Large ASF outbreak	
	SO1	SO2	LO1	LO2
Production	−0.044%	−0.044%	−7.33%	−7.33%
Exports	−20%	−40%	−40%	−80%

Source: Authors’ design.

Despite the historical precedent of trade restrictions, the U.S. position as one of the world’s largest pork suppliers could influence the scale and duration of these trade disruptions. Importing countries may weigh the economic and supply chain consequences of restricting U.S. pork, particularly given the global reliance on U.S. exports. In this context, the potential for regionalization, where trade is allowed to continue from unaffected, disease-free areas, could significantly limit the overall export losses. Studies have demonstrated that regionalization agreements can help mitigate the impacts of animal disease outbreaks, especially when containment measures are promptly implemented, and affected areas are clearly demarcated (39–41).

Given that the hypothetical small ASF outbreak in this analysis is assumed to be localized and contained, it is plausible to consider scenarios with relatively moderate export losses. The 20% decrease represents a case where regionalization is largely successful, allowing the unaffected regions of the U.S. to maintain trade. On the other hand, the 40% reduction reflects a more cautious scenario where some importing countries either delay recognizing regionalization agreements or impose stricter restrictions as a precautionary measure. These assumptions also account for the possibility of short-lived trade restrictions, as importing nations may resume trade relatively quickly if the U.S. demonstrates effective disease control and eradication measures. Regionalization has been successfully implemented in previous disease outbreaks. For example, during the 2014–2015 high pathogenic avian influenza (HPAI) outbreak, the U.S. established state- and county-level regionalization agreements. More recently, the U.S. has similar agreements with several trade partners, including South Korea, the Philippines, Qatar, and the United Kingdom [(42–44); USDA-FAS, (45, 46)]. Other examples include regionalization agreements for HPAI between China and France, as well as between the Philippines and the United Kingdom (47, 48).

To develop a plausible hypothetical scenario for a large ASF outbreak in the U.S., we drew on insights from the 2018–2019 ASF outbreak in China, one of the most severe outbreaks in history (7, 49, 50). The Chinese outbreak caused unprecedented losses in the swine industry, providing valuable data to inform potential production impacts in other major pork-producing countries. Using the reported swine loss data from You et al. (7), we estimated the potential reduction in U.S. hog and pork production over one year if a similar outbreak were to occur domestically. Our calculations indicate a 7.33% decline in U.S. hog and pork production within one year, corresponding to the elimination of approximately 5.5 million hogs. This level of production shock aligns closely with previous simulation studies, such as Paarlberg et al. (51), which estimated an 8.3% decline in the U.S. hog herd following a hypothetical Classical Swine Fever (CSF) outbreak.

For the large outbreak scenario, we modeled two levels of export losses to capture the potential range of trade impacts. The first scenario (LO1) assumes a 40% reduction in U.S. hog and pork exports, allowing for direct comparisons with the small outbreak scenario (SO2) where similar export shocks were considered (Table 1). The second scenario (LO2) assumes an 80% export reduction, reflecting the anticipated stronger policy responses and trade restrictions that would likely accompany a large-scale ASF outbreak in the U.S. This higher export loss scenario is based on the likelihood that importing nations would impose stricter and more prolonged trade bans to safeguard their domestic swine industries, particularly given the precedent set by China’s ASF outbreak, where trade disruptions were extensive and long-lasting (13, 52–54).

The assumptions in our study regarding partial trade bans due to a hypothetical ASF outbreak in the U.S. align more closely with recent international trade practices, which emphasize regionalization and targeted restrictions, unlike assumptions of long-lasting, complete trade bans [e.g., (12)]. Countries have increasingly adopted nuanced trade policies in response to ASF outbreaks, reflecting the need to balance biosecurity with market stability. For instance, during ASF outbreaks in Europe, importing countries targeted restrictions to affected regions rather than imposing blanket bans, minimizing disruptions to global pork supply chains (55, 56). Furthermore, the economic significance of U.S. pork exports, which accounted for nearly 16.2% of global exports in 2023 (57), would incentivize trade partners to limit the scope and duration of trade restrictions.

In the GTAP model, we introduce shocks to the technological parameters associated with U.S. hog and pork production and exports to capture both the direct impacts of the simulated scenarios and the economy-wide adjustments that arise from these changes. The GTAP model’s nested production structure, which is illustrated in Supplementary Figure A.1, provides a detailed framework for modeling these adjustments. Specifically, we apply shocks to the output-augmenting technical change parameter for sector 𝑗 in region 𝑟 ($\text{aoal}l_{j,r}$), representing the production decreases for each scenario. These shocks simulate the reduction in production efficiency or output levels due to the hypothetical ASF outbreak, ensuring that the model reflects the supply-side impacts on the U.S. hog and pork industries, while leaving output and factor demands endogenous so the model can reallocate resources and adjust prices in response. In the standard GTAP model, the sector-region-specific rate of output-augmenting technical change, $ao_{j,r}$, is the sum of a global sector component, a regional component, and the residual sector-region component we shock:


$$ao_{j,r}=\text{aose}c_{j}+\text{aore}g_{r}+\text{aoal}l_{j,r}$$ (1)

where $ao_{j,r}$ is the average rate of output augmenting technical change for sector j in region r; $\text{aose}c_{j}$ captures worldwide technical change specific to sector j; $\text{aore}g_{r}$ represents region-wide technical change affecting all sectors in region r; and $\text{aoal}l_{j,r}$ captures the sector-region-specific residual component. By construction, an exogenous shock to $\text{aoal}l_{j,r}$ maps into $ao_{j,r}$ and therefore affects production decisions throughout the top nest of the model’s production structure.

These shocks affect the model by changing firms’ demand for the bundle of core production inputs (labor, capital, and land), which are summarized as the “primary factor composite” (also called “value added,” meaning the part of output created by these basic inputs rather than by purchased intermediate goods). In the GTAP value-added demand equation, a decrease in $ao_{j,r}$ reduces productivity and shifts the derived demand for the primary factor composite, conditional on output and relative prices:


$$\mathrm{qv}a_{j,r}=-\mathrm{av}a_{j,r}+qo_{j,r}-ao_{j,r}-\text{ESUB}T_{j}\times (\begin{array}{l} \mathrm{pv}a_{j,r}-\mathrm{av}a_{j,r}\\ -ps_{j,r}-ao_{j,r} \end{array})$$ (2)

In Equation 2, $\mathrm{qv}a_{j,r}$ is the demand for primary factor composite in sector j, region r; $\mathrm{av}a_{j,r}$ is value-added augmenting technical change; $qo_{j,r}$ is sectoral output, $\text{ESUB}T_{j}$ represents the elasticity of substitution of intermediate inputs in production; $\mathrm{pv}a_{j,r}$ is the firms’ price of value added; and $ps_{j,r}$ is the supply price. Equations 1 and 2 show how the ASF production shock, implemented as a negative shock to $\text{aoal}l_{j,r}$, propagates through firms’ input demand and the broader equilibrium system.

Additionally, we represent ASF-related trade disruptions by shocking the import-augmenting technical change parameter ($\mathrm{am}s_{i,r,s}$) for hogs and pork (i) from the U.S. (r) into each destination region (s) in the model. In GTAP, bilateral trade flows are determined by the Armington structure, which treats goods as differentiated by origin. Within this framework, $\mathrm{am}s_{i,r,s}$ acts as a bilateral trade shifter: a negative shock to $\mathrm{am}s_{i,r,s}$ makes imports of product i from origin r effectively more “costly” (or less accessible) in destination s, capturing reduced market access due to sanitary restrictions, border measures, and related frictions. We calibrate the magnitude of these shocks so that they correspond to the scenario-specific percentage reductions in U.S. exports.

Under the Armington import-demand system, export sales of commodity i from region r to region s, $\mathrm{qx}s_{i,r,s}$, are linked to destination import demand and relative prices as follows:


$$\mathrm{qx}s_{i,r,s}=-\mathrm{am}s_{i,r,s}+\mathrm{qi}m_{i,s}-\text{ESUB}M_{i}\times (\begin{array}{l} \mathrm{pm}s_{i,r,s}-\mathrm{am}s_{i,r,s}\\ -\mathrm{pi}m_{i,s} \end{array})$$ (3)

where: $\mathrm{qx}s_{i,r,s}$ denotes exports of commodity i from region r to region s; $\mathrm{am}s_{i,r,s}$ represents the import-augmenting technical change of i from r into s; $\mathrm{qi}m_{i,s}$ is aggregate imports of i in region s at market prices; $\text{ESUB}M_{i}$ is a region-generic elasticity of substitution among imports of commodity i in the Armington structure; $\mathrm{pm}s_{i,r,s}$ is the domestic price for good i supplied from region r to region s; and $\mathrm{pi}m_{i,s}$ is market price of composite import i in region r.

By incorporating these export shocks, the model reflects both the immediate trade restrictions imposed by importing countries and the potential ripple effects on global pork markets. We keep production and export quantities as endogenous variables in the model, allowing them to adjust simultaneously in response to the exogenous shocks we simulate for production and trade. This approach ensures that the model captures additional adjustments arising from changes in factor use, intermediate inputs, and international trade flows, thereby providing a broad representation of the potential economic impacts of the ASF outbreak scenarios.

In interpreting these shocks, it is important to emphasize that we treat ASF-related mortality and depopulation as physical eliminations from the herd and, consequently, from pork supply. That is, eliminated animals are not assumed to enter any market channel and are therefore not available for export or domestic consumption. This physical loss is represented exclusively through the negative production shock implemented via output-augmenting technical change, which reduces total sectoral output. By contrast, the bilateral trade shock implemented through import-augmenting technical change is not a physical supply reduction; it is a representation of sanitary trade restrictions and market-access frictions that lower foreign demand for U.S.-origin pork relative to other sources. Because domestic production and bilateral trade flows are endogenous in the model, GTAP determines how the reduced supply is allocated between domestic absorption and exports given the export restrictions.

## 3 Results

**Figure 1:**
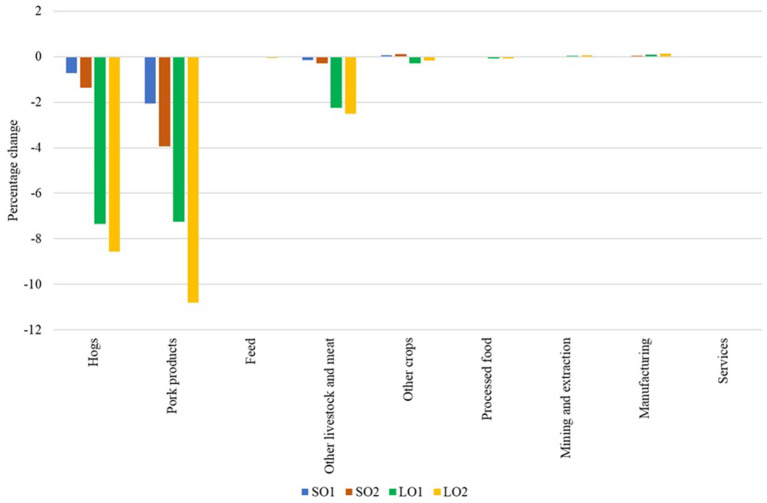
Percentage changes in U.S. output. Source: Authors’ simulations. Scenario SO1 considers a hog and pork production decrease of 0.044% and an export loss of 20%. Scenario SO2 assumes the same production decrease, but considers an export loss of 40%. Scenario LO1 combines a hog and pork production decrease of 7.33% with an export loss of 40%. Scenario LO2 represents the worst-case scenario, with a decrease in hog and pork production of 7.33% and an export loss of 80%.

Figure 1 illustrates the simulated impacts of hypothetical ASF outbreaks on production across different sectors of the U.S. economy. In the small outbreak scenarios (SO1 and SO2), the impacts are relatively minor, with the most substantial changes occurring in the hog and pork sectors as expected. Hog production decreases by 0.71% in SO1 and 1.37% in SO2, while pork production decreases by 2.05% in SO1 and 3.94% in SO2. Other sectors show minimal changes. However, in the large outbreak scenarios (LO1 and LO2), the impacts are more pronounced. Hog production decreases by 7.34% in LO1 and 8.57% in LO2, while pork production decreases by 7.25 and 10.8%, respectively. The larger decrease in hog and pork output leads to a 2.24% decrease in other livestock and meat production in LO1, and a 2.51% decrease in LO2. The remaining sectors have smaller simulated changes. These results show that a small ASF outbreak as modeled might be manageable with minimal economic disruption across sectors, while a large outbreak could severely impact the U.S. pork industry and negatively affect related industries, potentially leading to shortages and higher prices.

**Figure 2:**
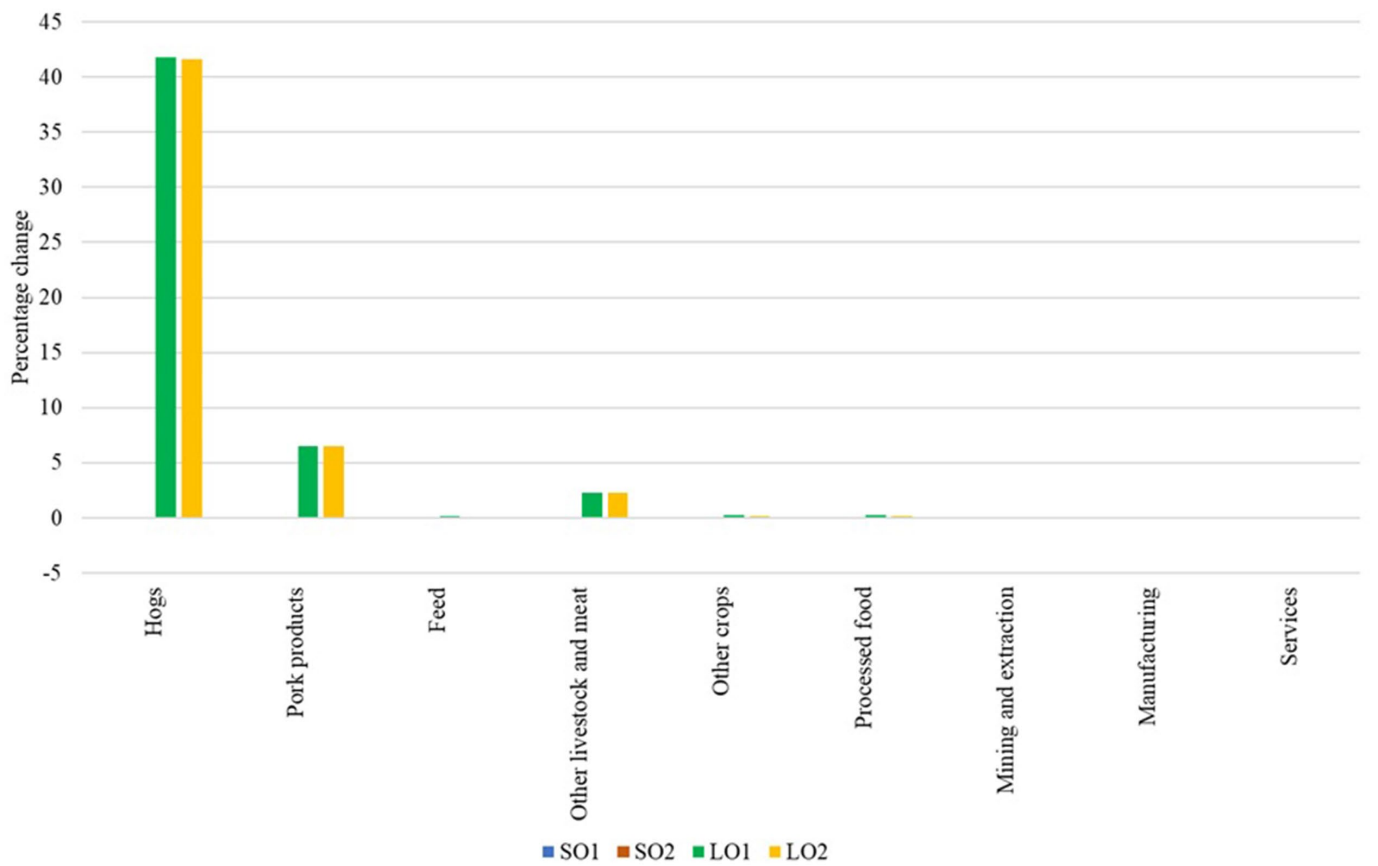
Percentage changes in U.S. producer prices. Source: Authors’ simulations. Scenario SO1 considers a hog and pork production decrease of 0.044% and an export loss of 20%. Scenario SO2 assumes the same production decrease, but considers an export loss of 40%. Scenario LO1 combines a hog and pork production decrease of 7.33% with an export loss of 40%. Scenario LO2 represents the worst-case scenario, with a decrease in hog and pork production of 7.33% and an export loss of 80%.

Figure 2 shows the simulated changes in U.S. producer prices. In the small outbreak scenarios, hog prices decrease slightly (0.01% in SO1 and 0.06% in SO2), while pork prices increase marginally (0.04% in SO1 and 0.02% in SO2). However, in the large outbreak scenarios, hog prices increase dramatically (41.78% in LO1 and 41.60% in LO2), and pork prices also present a substantial increase (6.55% in LO1 and 6.50% in LO2). The other livestock and meat sector shows a price increase of 2.30% on average in the large outbreak scenarios. Other sectors show smaller price changes. The substantial increase in hog prices during a large outbreak could lead to significant financial strain for pork processors and consumers. The similar price outcomes in LO1 and LO2, despite differing export shocks, suggest that domestic production losses dominate the price dynamics in these scenarios. The 7.33% reduction in U.S. hog and pork production shifts the domestic supply curve inward substantially, raising marginal costs and tightening availability. While a larger export restriction reduces foreign demand for U.S.-origin pork, the change in demand from a 40% to an 80% restriction is limited in its effect on the domestic producer price. Under GTAP’s Armington structure, the export restriction is implemented as a bilateral trade friction, which changes the destination mix of sales and induces trade diversion and domestic absorption rather than creating an additional physical supply expansion. As a result, the larger export restriction primarily affects quantities traded and the allocation of the reduced output across destinations, while equilibrium producer prices remain anchored by the supply shock. Consistent with this mechanism, differences between LO1 and LO2 are more visible in bilateral trade flows and destination market outcomes than in U.S. producer prices.

**Figure 3:**
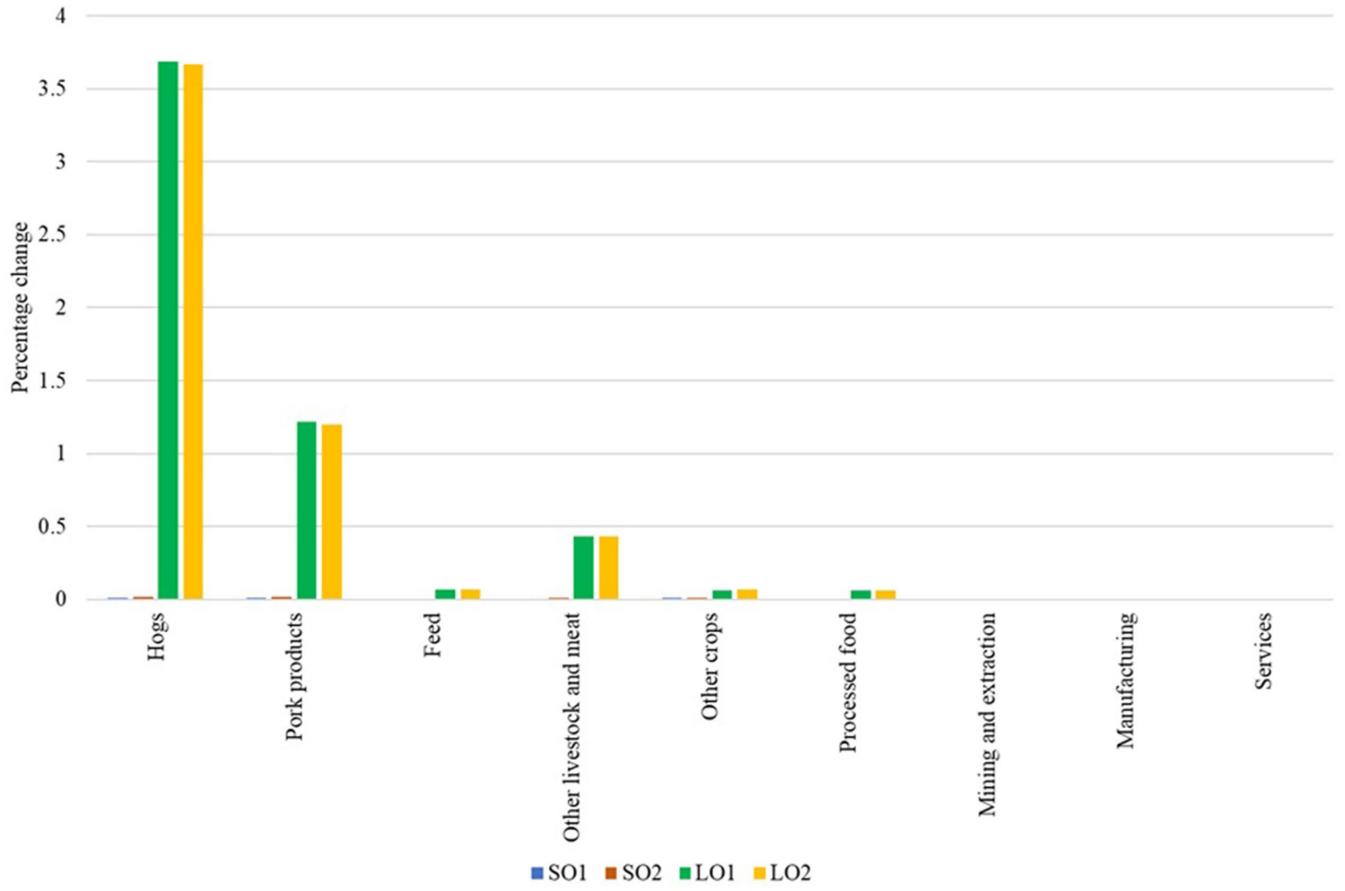
Percentage changes in world prices. Source: Authors’ simulations. Scenario SO1 considers a hog and pork production decrease of 0.044% and an export loss of 20%. Scenario SO2 assumes the same production decrease, but considers an export loss of 40%. Scenario LO1 combines a hog and pork production decrease of 7.33% with an export loss of 40%. Scenario LO2 represents the worst-case scenario, with a decrease in hog and pork production of 7.33% and an export loss of 80%.

The small outbreak scenarios show minimal impacts on world prices, with negligible increases in hog and pork prices, while other sectors’ prices are unaffected (Figure 3). The large outbreak scenarios, on the other hand, lead to more noteworthy changes in world prices, particularly for hog prices (3.69% increase in LO1 and 3.67% in LO2) and pork prices (1.22% increase in LO1 and 1.20% in LO2). Other sectors show negligible or no price changes across all four scenarios. The other livestock and meat sector also shows a simulated increase of 0.43% in prices worldwide in both large outbreak scenarios. In general, a small ASF outbreak in the U.S. would not have noticeable effects on global pork markets, however, a large outbreak could substantially disrupt global pork supply chains, potentially leading to price volatility in international markets. Results show that non-agricultural sectors do not show price changes, independent of the size of the outbreak.

Considering all four scenarios, global pork exports decrease between 1.41% (SO1) and 4.16% (LO2). Supplementary Tables A.1–A.8 show changes in hog and pork bilateral trade for each outbreak scenario. The U.S. experiences the most substantial hog and pork export declines across all scenarios, consistent with the imposed shocks in the model. A comparison of the scenarios reveals distinct differences in the scale of trade realignments. The small outbreak scenarios (SO1 and SO2) result in limited changes in global trade patterns, reflecting the relatively minor disruptions caused by these scenarios. However, the large outbreak scenarios (LO1 and LO2) demonstrate a significant redistribution of export shares. The transition from SO2 to LO1 is particularly noteworthy, as both scenarios assume a − 40% export shock but differ in the production shocks applied. The larger production shock in LO1 (−7.33%) amplifies the trade realignment, leading to more pronounced increases in exports from competing regions.

Our modeling results show that other competing countries would increase exports to fill the gap left by reduced U.S. exports. Import patterns change as countries adjust to the new trade landscape. Canada and Mexico, especially, increase hog and pork imports from other countries. Supplementary Tables A.1–A.4 show that Mexico increases hog imports from sources other than the U.S. relatively more than Canada, while Supplementary Tables A.5–A.8 show that Canada increases pork imports from other sources relatively more than Mexico. In general, the results reveal complex adjustments in global pork trade flows in response to the hypothetical ASF outbreaks in the U.S., highlighting the interconnected nature of global agricultural markets.

In all scenarios, the substantial decrease in U.S. hog and pork exports triggers shifts in trade flows as other countries increase exports to fill the gap, with particularly pronounced adjustments in the large outbreak scenarios (LO1 and LO2). However, these adjustments are not costless. Exporting nations may face increased production costs as they expand output, and importing nations may encounter higher prices as they source pork from more expensive or less geographically proximate suppliers. The simulated increase in global pork prices (1.22% in LO1 and 1.20% in LO2) reflects these adjustments and highlights the challenges of maintaining price stability in the face of major supply shocks in a key global export market.

The bilateral trade dynamics revealed by this analysis underscore the intricate nature of global pork markets and the potential for realignment in the event of an ASF outbreak in the U.S. The simulated changes in trade patterns indicate a likely scenario where other pork-exporting nations have the opportunity to expand their market presence, potentially securing increased market share and establishing new trade relationships. This trade diversion could have lasting implications for the global pork industry conjuncture. Furthermore, the nuanced changes in import behaviors, particularly the responses by Canada and Mexico, emphasize the critical importance of regional trade interdependencies within North America.

In the small outbreak scenarios, SO1 and SO2, hypothetical ASF outbreaks in the U.S. have no discernible effects on the gross domestic product (GDP) of the U.S. or other countries and regions. However, the large outbreak scenarios lead to a small simulated decrease in U.S. GDP (−0.05% in both LO1 and LO2), with negligible effects on GDP in other regions. To put the U.S. effects into perspective, a large ASF outbreak could lead to a $12.72 billion loss, considering the U.S. GDP in 2022.

Table 2 presents welfare impacts of hypothetical ASF outbreaks in monetary terms. The U.S. experiences simulated welfare losses of $310 million in SO1 and $563 million in SO2. In the case of a large outbreak, the U.S. faces substantial simulated welfare losses, with a $10.9 billion loss in LO1 and a $11.4 billion loss in LO2. In other words, a large outbreak, combined with a sharp decrease in pork exports, could potentially lead to a national welfare loss in the U.S. 38 times larger than in a small outbreak scenario. Other countries have relatively small economic welfare changes in both scenarios as countries substitute import sourcing away from the U.S. towards other suppliers. China and Hong Kong, the rest of Asia and Oceania, South and Southeast Asia, and Central and Southern Africa present simulated welfare losses for both a small and large ASF outbreak in the U.S.

**Table 2:** Changes in welfare (equivalent variation, $US million).

Country	SO1	SO2	LO1	LO2
USA	−310.0	−563.0	−10,905.0	−11,413.0
Canada	45.1	90.5	320.0	423.0
Mexico	24.1	55.9	120.0	207.0
Brazil	10.3	31.1	154.0	216.0
China and Hong Kong	−31.1	−54.9	−72.0	−111.0
Rest of Asia and Oceania	−108.0	−191.0	−349.0	−480.0
South and Southeast Asia	−18.0	−32.9	−77.1	−103.0
Central and South America	1.7	2.1	61.7	61.2
Europe	62.5	109.0	141.0	221.0
Middle East and North Africa	44.8	69.9	−3.6	21.3
Central and Southern Africa	−9.7	−18.6	−21.8	−38.6

Source: Authors’ simulations.

Scenario SO1 considers a hog and pork production decrease of 0.044% and an export loss of 20%. Scenario SO2 assumes the same production decrease, but considers an export loss of 40%. Scenario LO1 combines a hog and pork production decrease of 7.33% with an export loss of 40%. Scenario LO2 represents the worst-case scenario, with a decrease in hog and pork production of 7.33% and an export loss of 80%.

**Figure 4:**
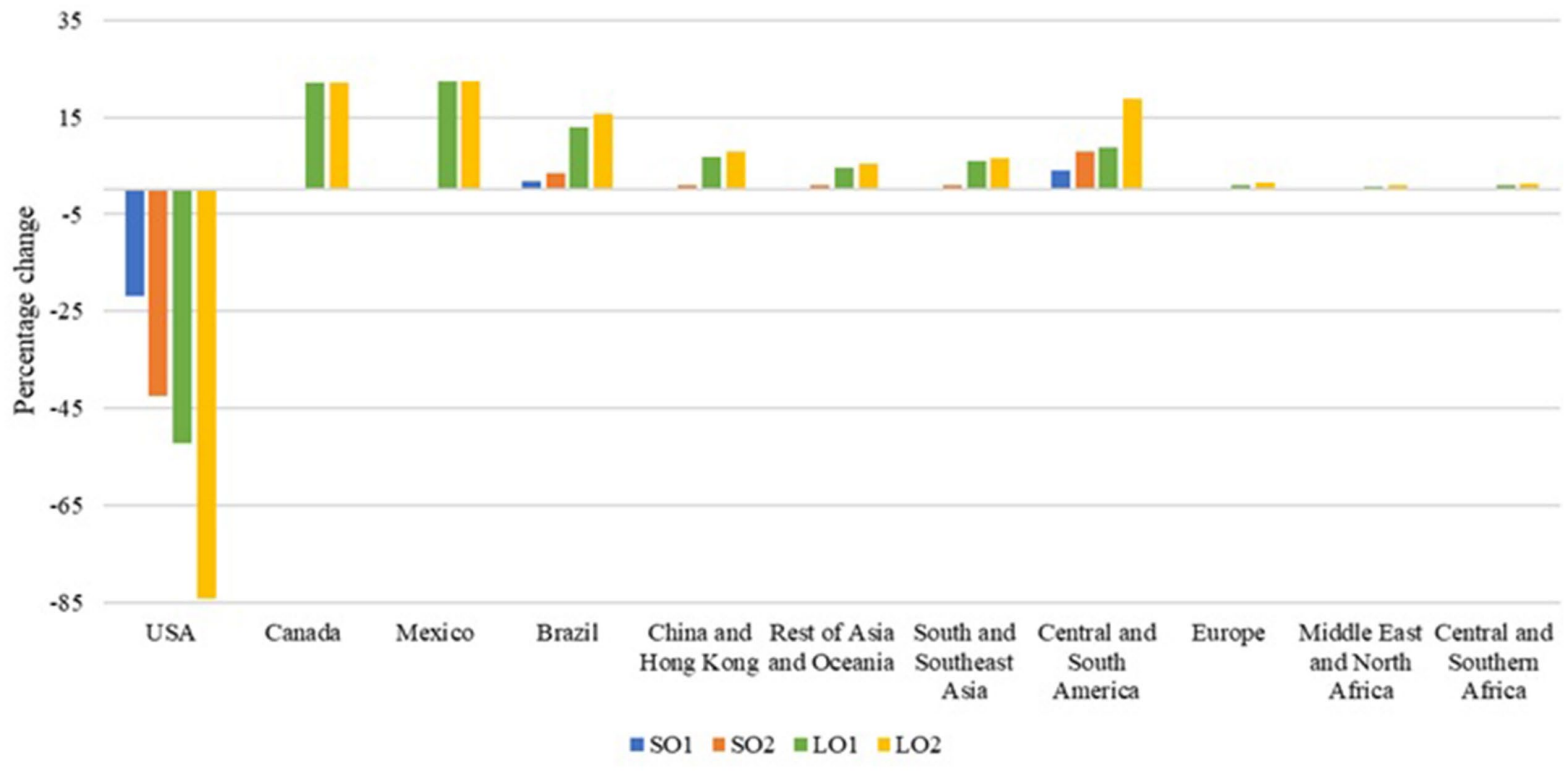
Percentage changes in hog exports. Source: Authors’ simulations. Scenario SO1 considers a hog and pork production decrease of 0.044% and an export loss of 20%. Scenario SO2 assumes the same production decrease, but considers an export loss of 40%. Scenario LO1 combines a hog and pork production decrease of 7.33% with an export loss of 40%. Scenario LO2 represents the worst-case scenario, with a decrease in hog and pork production of 7.33% and an export loss of 80%.

**Figure 5:**
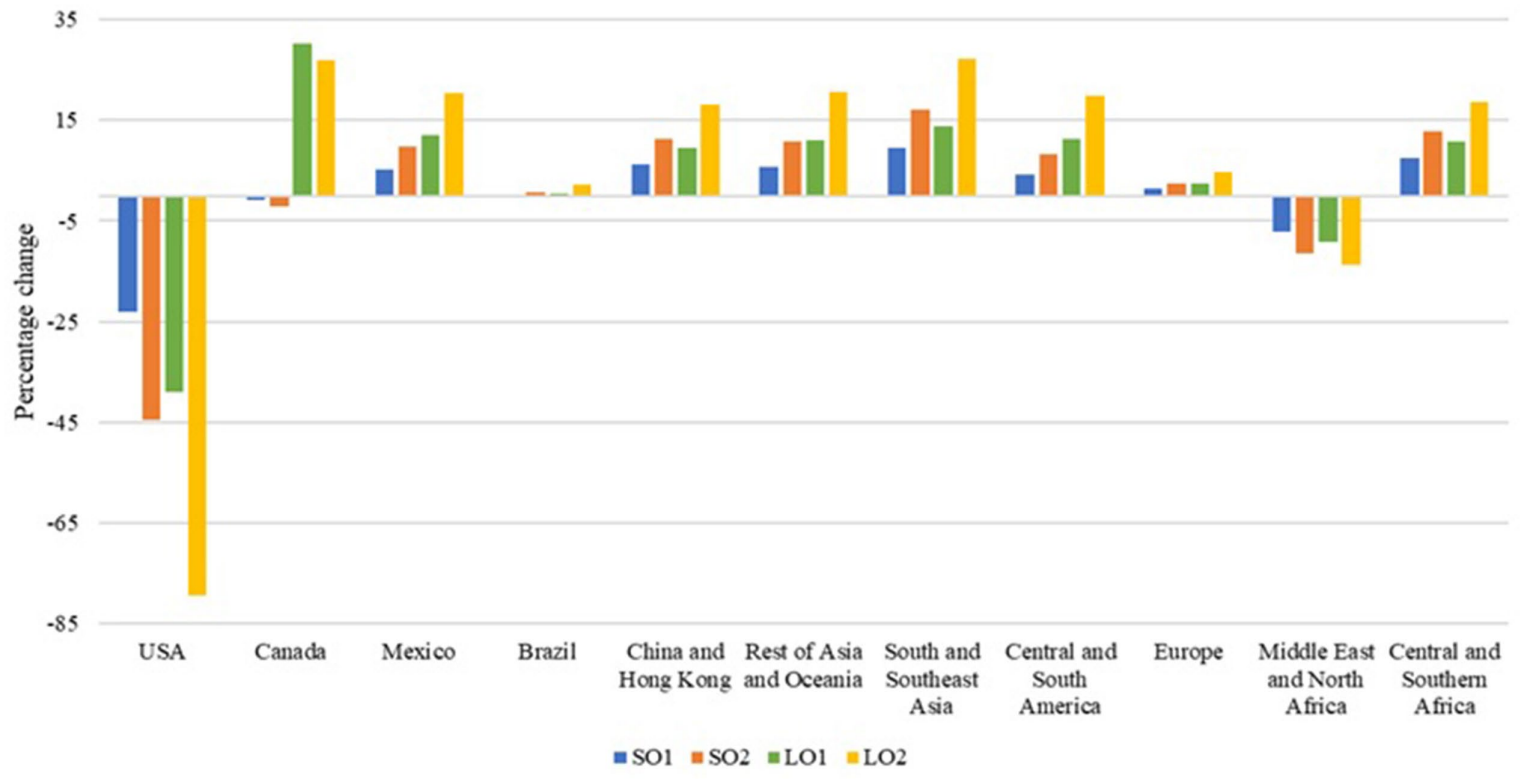
Percentage changes in pork exports. Source: Authors’ simulations. Scenario SO1 considers a hog and pork production decrease of 0.044% and an export loss of 20%. Scenario SO2 assumes the same production decrease, but considers an export loss of 40%. Scenario LO1 combines a hog and pork production decrease of 7.33% with an export loss of 40%. Scenario LO2 represents the worst-case scenario, with a decrease in hog and pork production of 7.33% and an export loss of 80%.

Canada, Europe, Mexico, and Brazil benefit in terms of economic welfare from both a small and a large outbreak, reflecting trade diversion away from U.S. products and toward alternative suppliers. This adjustment is visible in the simulated export responses. Under the large outbreak scenarios, competing exporters expand hog exports (Figure 4) by 22.2% (Canada) and 22.5% (Mexico) in both LO1 and LO2. Brazil also expands hog exports by 13.0% in LO1 and 15.8% in LO2, while Europe exhibits smaller increases (0.9% in LO1; 1.4% in LO2). A similar pattern holds for pork exports (Figure 5): in LO1, pork exports rise by 30.2% in Canada, 12.0% in Mexico, 2.6% in Europe, and 0.5% in Brazil; in LO2, pork exports increase by 26.9% in Canada, 20.3% in Mexico, 4.7% in Europe, and 2.3% in Brazil. These export expansions are indicatives of the welfare gains for these regions (Table 2), which benefit from favorable price and trade adjustments following the contraction in U.S. supply and exports.

Supplementary Tables A.9–A.12 present the decomposition of welfare changes, measured in equivalent variation (in millions of US dollars), for countries and regions under small and large ASF outbreak scenarios in the U.S. The welfare effects are broken down into five components: aggregate welfare effect, allocative efficiency contribution, technological change contribution, terms of trade contribution, and savings and investment contribution. In the small outbreak scenario SO1, the U.S. experiences a negative aggregate welfare effect of $310 million, primarily driven by a deterioration in the terms of trade equivalent to -$257 million (Supplementary Table A.9). In SO2, the $563-million welfare loss in the U.S. is also mostly caused by a decrease in the terms of trade, corresponding to -$499 million (Supplementary Table A.10). On the other hand, in the large outbreak scenarios, the U.S. welfare losses are mainly driven by large negative technological change contributions, which lead to a welfare loss of approximately $10 billion in both LO1 and LO2 (Supplementary Tables A.11, A.12). Most other countries face small negative or positive welfare effects. Terms of trade contributions are generally positive for other countries, indicating they benefit from changes in relative prices. Several countries, including Canada, Mexico, Brazil, and Europe, experience positive aggregate welfare effects. The allocative efficiency and terms of trade contributions vary widely across countries, reflecting complex adjustments in global markets.

The economic welfare decomposition for the small outbreak scenarios indicates that the U.S. economy demonstrates a degree of resilience to minor shocks in the pork industry as simulated for this analysis. The relatively modest economic welfare losses suggest that the effects of small-scale ASF outbreaks could be localized and manageable, provided that effective containment measures are implemented swiftly. On the other hand, the decomposition for large outbreak scenarios highlights the severe potential negative impacts on U.S. welfare, driven primarily by significant losses in productive capacity and adverse terms-of-trade effects if countries impose trade restrictions. The negative technological change contribution reflects the loss of productive capacity in the pork sector. These findings underscore the disproportionate economic consequences of large-scale outbreaks, particularly for industries as globally integrated as pork supply chains.

### 3.1 Sensitivity analysis

To assess the robustness of the model results, this research employs a systematic sensitivity analysis (SSA), a standard approach in CGE modeling for evaluating uncertainty. The first step of the SSA involves testing the sensitivity of the results to variations in shock values by specifying a range of plausible values for each shock. Within this range, we estimate means and standard deviations, enabling the calculation of confidence intervals for the model results. For this purpose, we use Chebyshev’s theorem, which is particularly suitable as it does not require any assumptions about the probability distribution of the results for each variable (23).

The SSA in this study focuses primarily on variations in shocks related to production and export losses, with shocks increased and decreased by 50%. Additionally, we extend the analysis to test the sensitivity of results to variations in the elasticity of substitution between domestic and imported hogs and pork. By exploring a range of elasticity values, we systematically evaluate how uncertainty in these key parameters influences the results. This tool draws on established practices in CGE modeling to address uncertainty in exogenous variables systematically. Specifically, the inclusion of elasticity variations accounts for potential discrepancies in how domestic and imported goods are substituted under different scenarios, a critical factor in trade-focused analyses (58, 59).

Table 3 summarizes the SSA results for U.S. welfare across the four ASF scenarios. In SO1, the economic welfare losses range between -$431 million and -$187 million, while, in SO2, the range expands to -$764 million and -$354 million. This progression demonstrates the increasing severity of economic welfare losses with larger export shocks. LO1 results in an economic welfare loss with a wide confidence interval spanning a potential loss of -$25.2 billion to a gain of $2.9 billion. LO2, which introduces even larger export shocks, presents a confidence interval ranging from a loss of -$25.4 billion to a gain of $2.2 billion. These wide bounds are a direct consequence of two factors: the large magnitude of the export shocks considered and the conservative nature of Chebyshev’s inequality. Importantly, the positive upper bounds do not represent typical or likely outcomes, but rather extreme equilibrium cases in which very large reductions in exports generate sharp declines in domestic pork prices, leading to strong increases in domestic consumption and, in turn, temporary gains in measured economic welfare. From a policy perspective, the key result of the SSA is the robustness of the central finding: across a broad and conservative range of shock magnitudes, large ASF outbreaks are associated with substantial expected welfare losses for the U.S. The baseline and median outcomes for LO1 and LO2, approximately $11 billion in welfare losses, lie well within the interior of the SSA bounds and remain strongly negative. The SSA therefore reinforces the conclusion that large ASF outbreaks pose significant economic risks, even when accounting for uncertainty in exogenous shocks. The SSA results for the elasticity of substitution between domestic and imported hogs and pork in the U.S. examines how changes in this parameter influence economic welfare outcomes. Increasing the elasticity of substitution by 50% reflects a scenario where consumers and firms can more easily switch between domestic and imported goods. For example, if the price of imported goods rises, demand adjusts more readily to domestically produced substitutes. Conversely, decreasing the elasticity by 50% assumes greater rigidity in switching between goods, meaning that even with price changes, consumers and firms are less likely to adjust their preferences.

**Table 3:** SSA with variation in shocks to production and exports, 95% confidence interval.

Shocked variable		Shock range	SO1		
			U.S. welfare change (in million $US)		
			Model result	Lower bound	Upper bound
Production	Hogs	[−0.066%; −0.022%]	−310	−431	−187
	Pork				
Exports	Hogs	[−10%; −30%]			
	Pork				

Shocked variable		Shock range	SO2		
			U.S. welfare change (in million $US)		
			Model result	Lower bound	Upper bound
Production	Hogs	[−0.066%; -0.022%]	−563	−764	−354
	Pork				
Exports	Hogs	[−20%; -60%]			
	Pork				

Shocked variable		Shock range	LO1		
			U.S. welfare change (in million $US)		
			Model result	Lower bound	Upper bound
Production	Hogs	[−11.00; −3.67%]	−10,905	−25,182	2,113
	Pork				
Exports	Hogs	[−20%; −60%]			
	Pork				

Shocked variable		Shock range	LO2		
			U.S. welfare change (in million $US)		
			Model result	Lower bound	Upper bound
Production	Hogs	[−11.00; −3.67%]	−11,413	−25,445	2,182
	Pork				
Exports	Hogs	[−40%; −100%]			
	Pork				

Source: Authors’ simulations.

The results in Table 4 highlight how these changes affect U.S. economic welfare under different scenarios (SO1, SO2, LO1, and LO2). In SO1 and SO2, the economic welfare losses range from -$581 million to -$36 million, and from -$1 billion to -$98 million. The lower and upper bounds vary more widely, especially in SO2, reflecting greater sensitivity to elasticity changes under larger export shocks. For the more severe shock scenarios, LO1 and LO2, the economic welfare impacts are significantly larger, with model results showing losses between -$11.4 billion and -$10.4 billion, and from -$12 billion to -$10.8 billion, respectively. The confidence intervals for these scenarios are much narrower compared to SO1 and SO2. These tighter bounds suggest less variability in outcomes despite the severity of the shocks, indicating that substitution elasticity plays a relatively smaller role in these extreme scenarios compared to the larger magnitude of production and export shocks.

**Table 4:** SSA with variation in elasticity of substitution for hog and pork sectors, 95% confidence interval.

SO1			SO2		
U.S. welfare change (in million $US)			U.S. welfare change (in million $US)		
Model result	Lower bound	Upper bound	Model result	Lower bound	Upper bound
−310	−581	−36	−563	−1,014	−98

LO1			LO2		
U.S. welfare change (in million $US)			U.S. welfare change (in million $US)		
Model result	Lower bound	Upper bound	Model result	Lower bound	Upper bound
−10,905	−11,380	−10,422	−11,413	−11,962	−10,817

Source: Authors’ simulations.

## 4 Discussion

This study reveals the complex and far-reaching consequences of a potential ASF outbreak in the U.S. Our results show that even without assuming a complete suspension of U.S. hog and pork exports, as in Carriquiry et al. (12), partial export restrictions can generate substantial welfare losses for the U.S. and non-trivial impacts for U.S. trade partners. In large outbreak scenarios, simulated U.S. welfare losses exceed $10 billion, primarily driven by production disruptions and adverse terms-of-trade effects. Import-dependent regions, particularly in Asia and Africa, experience welfare losses as they adjust to reduced U.S. supplies and higher world prices, while major competing exporters, such as Brazil, Canada, Mexico, and European countries, gain by expanding exports and capturing market share. This redistribution of economic welfare highlights how shocks originating in a single large exporter propagate through global pork markets, reinforcing that animal disease events are not merely sectoral disturbances but economic-wide shocks. These results are consistent with broader evidence on how major disease shocks reconfigure agricultural trade and prices (13, 20, 27, 39).

A central policy implication of these findings is that the economic costs of delayed detection and containment rise nonlinearly with outbreak size. The stark contrast between the small and large outbreak scenarios indicates that policies capable of preventing escalation, such as early detection systems, rapid depopulation protocols, and movement controls, generate benefits far beyond the livestock sector. From an economic perspective, these measures function as loss-avoidance investments: preventing a large outbreak avoids welfare losses an order of magnitude larger than those associated with small, localized events. This provides a clear economic justification for sustained public investment in surveillance, diagnostics, and response capacity, complementing existing animal-health rationales (15, 42, 51).

Beyond outbreak control, the results highlight the critical role of trade policy design in shaping economic outcomes. Even when production losses are held constant, differences in export restrictions generate substantial variation in welfare losses. This finding underscores the economic value of regionalization and zoning agreements that allow disease-free areas to continue exporting. Our simulations show that scenarios consistent with partial rather than extreme trade bans substantially reduce welfare losses for the U.S. while limiting global price increases and trade diversion. These results support policies aimed at strengthening pre-negotiated regionalization agreements and improving transparency and trust with trade partners, consistent with evidence from HPAI occurrences [(41, 42); WTO, 2018].

The redistribution of welfare across regions has important international implications. Import-dependent regions, particularly in Asia and Africa, experience welfare losses driven by higher world prices and reduced access to U.S. pork, while major exporters benefit from expanded market opportunities. These asymmetric effects suggest that ASF outbreaks in the U.S. have consequences for global food security and trade stability, especially for low- and middle-income importing countries with limited capacity to absorb price shocks. From a policy standpoint, this reinforces the importance of international coordination, including information sharing, harmonized sanitary standards, and contingency planning to mitigate spillovers from disease outbreaks in major exporting countries.

The magnitude of simulated economic losses also has implications for public budgeting and institutional design. Losses on the scale estimated in large outbreak scenarios outweigh typical annual expenditures on animal-health surveillance and preparedness, implying high benefit–cost ratios for preventive investments. Moreover, because welfare losses are driven not only by production declines but also by trade disruptions, effective ASF preparedness requires coordination across agricultural, trade, and foreign-affairs institutions. Policies narrowly focused on domestic disease control, without parallel efforts to preserve market access, risk leaving substantial economic value unrealized.

Methodologically, this study illustrates the value of a global CGE framework for evaluating the economy-wide implications of transboundary animal diseases. By explicitly modeling production, consumption, factor markets, and bilateral trade flows, the GTAP-based approach captures interactions that partial equilibrium and input–output models may miss, such as ripple effects across sectors and markets and general equilibrium adjustments in prices and income. In doing so, this research complements existing ASF work that has focused more narrowly on sectoral or consumer outcomes, and aligns with previous CGE studies of other livestock diseases. At the same time, the framework provides a robust structure for testing alternative policy scenarios and conducting systematic sensitivity analysis.

The analysis also points to several avenues for future research. First, integrating epidemiological models that explicitly capture outbreak duration and spatial spread would allow policymakers to evaluate time-dependent tradeoffs between containment costs and avoided economic losses. Dynamic disease-economic models could capture time paths of inventory rebuilding, investment responses, and gradual recovery of trade flows. Second, distributional analyses within the U.S. and key trade partners, including heterogeneous impacts on producers by size, region, and integration into value chains, would complement the aggregate welfare results presented here and provide more targeted guidance for compensation and adjustment policies.

ASF preparedness should be treated not only as an animal health priority but as a macroeconomic and trade-policy concern. By quantifying how alternative combinations of outbreak severity and trade restrictions propagate through production, prices, trade flows, and welfare, this research provides insights for risk assessment and policy design for ASF and other transboundary livestock diseases. The potential for multi-billion-dollar welfare losses in large outbreak scenarios offers a strong economic justification for continued investment in disease prevention, surveillance, and rapid response capacity, as well as trade agreements that can limit export disruptions. Overall, this study illustrates the value of economic modeling in helping decision-makers prioritize resources and design policies to prepare for potential animal health crises.

## Data Availability

The analysis is based on proprietary data from the Global Trade Analysis Project (GTAP) database. Requests to access these datasets should be directed to https://www.gtap.agecon.purdue.edu/databases/default.asp.

## References

[1] Juszkiewicz M Walczak M Woźniakowski G Podgórska K. African swine fever: transmission, spread, and control through biosecurity and disinfection. Includ Polish Trends Viruses. (2023) 15:2275. doi: 10.3390/v15112275, 38005951 PMC10674562

[2] Beltrán-Alcrudo D. Arias M. Gallardo C. Kramer S. Penrith M.K. 2017. African Swine Fever: Detection and Diagnosis. Food and Agriculture Organization of the United Nations (Rome:FAO). Available online at https://openknowledge.fao.org/server/api/core/bitstreams/d6d6ad7b-b4d7-4116-a95e-06826e8a25b0/content (Accessed January 22, 2025).

[3] Fernandez-Colorado CP Kim WH Flores RA Min W. African swine fever in the Philippines: a review on surveillance, prevention, and control strategies. Animals. (2024) 14:1816. doi: 10.3390/ani14121816, 38929435 PMC11200829

[4] Ruiz-Saenz J Diaz A Bonilla-Aldana DK Rodriguez-Morales AJ Martinez-Gutierrez M Aguilar PV. African swine fever virus: a re-emerging threat to the swine industry and food security in the Americas. Front Microbiol. (2022) 13:1011891. doi: 10.3389/fmicb.2022.1011891, 36274746 PMC9581234

[5] USDA. U.S. Department of Agriculture. (2023). African swine fever response plan: The red book. Foreign animal disease preparedness & response plan. Available online at: https://www.aphis.usda.gov/sites/default/files/asf-responseplan.pdf (Accessed January 22, 2025).

[6] Yao H Zang C Zuo X Xian Y Lu Y Huang Y . Tradeoff analysis of the pork supply and food security under the influence of African swine fever and the COVID-19 outbreak in China. Geogr Sustain. (2022) 3:32–43. doi: 10.1016/j.geosus.2022.01.005

[7] You S Liu T Zhang M Zhao X Dong Y Wu B . African swine fever outbreaks in China led to gross domestic product and economic losses. Nat Food. (2021) 2:802–8. doi: 10.1038/s43016-021-00362-1, 37117973

[8] Jean-Pierre RP Hagerman AD Rich KM. An analysis of African swine fever consequences on rural economies and smallholder swine producers in Haiti. Front. Vet. Sci. (2022) 9:960344. doi: 10.3389/fvets.2022.960344, 36311651 PMC9597192

[9] Brazilian Ministry of Agriculture and Livestock. (2022). Plano de contingência para Peste Suína Africana. Available online at: https://www.gov.br/agricultura/pt-br/assuntos/sanidade-animal-e-vegetal/saude-animal/programas-de-saude-animal/sanidade-suidea/legislacao-suideos/Plano_Contingencia_PSA__versao_1.0_15_09_2022_final.pdf (Accessed January 23, 2025).

[10] Ferry E. Thompson D. Zangaro C.. (2022). African swine fever outbreak in the Dominican Republic spurs the need for enhanced biosecurity on swine farms in the U.S. Michigan State University extension. Available online at https://www.canr.msu.edu/news/african-swine-fever-outbreak-in-the-dominican-republic-spurs-the-need-for-enhanced-biosecurity-on-swine-farms-in-the-u-s (Accessed December 4, 2024).

[11] WOAH. World Organization for Animal Health. (2021). Self-declaration of the establishment of a protection zone for U.S. territories in the Caribbean. Available online at: https://www.woah.org/app/uploads/2021/10/2021-10-usa-asf-pz-uscaribbean.pdf (Accessed January 22, 2025).

[12] Carriquiry M. Elobeid A. Swenson D. Hayes D.. (2021). Analysis of an African swine fever outbreak in the United States: Implications on National and Iowa Agriculture. In: 2021 Agricultural and Applied Economics Association Annual Meeting, August 1–3, Austin, TX.

[13] Gale F. Kee J. Huang J.. (2023). How China’s African swine fever outbreaks affected global pork markets. Economic Research Service, Economic Research Report no. 326.

[14] Herrera-Ibatá DM Martínez-López B Quijada D Burton K Mur L. Quantitative approach for the risk assessment of African swine fever and classical swine fever introduction into the United States through legal imports of pigs and swine products. Plos One. (2017) 12:e0182850. doi: 10.1371/journal.pone.0182850, 28797058 PMC5552331

[15] McKee SC Brown VR Shwiff SA Giallombardo GM Miller RS. Areas within the United States at the highest risk for African swine fever, classical swine fever, and foot-and-mouth disease introduction. Transbound Emerg Dis. (2023) 2023:8892037. doi: 10.1155/2023/8892037, 40303669 PMC12016723

[16] Rendleman CM Spinelli FJ. The costs and benefits of animal disease prevention: the case of African swine fever in the US. Environ Impact Assess Rev. (1999) 19:405–26. doi: 10.1016/S0195-9255(99)00016-5

[17] Lee J Schulz LL Hoffman E Tonsor GT. How can reporting on foreign animal diseases affect meat purchases? The case of African swine fever. J Agric Resour Econ. (2022) 52:89–111. doi: 10.1017/age.2022.23

[18] Kashyap P Suter JF McKee SC. Measuring changes in pork demand, welfare effects, and the role of information sources in the event of an African swine fever outbreak in the United States. Food Policy. (2024) 126:102672. doi: 10.1016/j.foodpol.2024.102672

[19] Ahmed L.. (2020). Impacts of Chinese African swine fever losses and rebuilding on the U.S. agricultural sector. 23rd annual Conference on Global Economic Analysis (Virtual Conference). Available online at: https://ageconsearch.umn.edu/record/333162/?v=pdf (Accessed January 22, 2025).

[20] Mason-D’Croz D Bogard JR Herrero M Robinson S Sulser TB Wiebe K . Modelling the global economic consequences of a major African swine fever outbreak in China. Nat Food. (2020) 1:221–8. doi: 10.1038/s43016-020-0057-2, 33634268 PMC7116817

[21] Hertel T. Global Trade Analysis: Modeling and Applications. Cambridge: Cambridge University Press (1997).

[22] Corong E Hertel T McDougall R Tsigas M van der Mensbrugghe D. The standard GTAP model, version 7. J Glob Econ Anal. (2017) 1:1–119. doi: 10.21642/JGEA.020101AF

[23] Burfisher ME. Introduction to Computable General Equilibrium Models. 3rd ed. Cambridge: Cambridge University Press (2020).

[24] Boisvert RN Kay D Turvey CG. Macroeconomic costs to large scale disruptions of food production: the case of foot- and-mouth disease in the United States. Econ Model. (2012) 29:1921–30. doi: 10.1016/j.econmod.2012.06.007

[25] Countryman AM Hagerman AD. Retrospective economic analysis of foot and mouth disease eradication in the Latin American beef sector. Agribusiness. (2017) 2:257–73. doi: 10.1002/agr.21472

[26] De Menezes TC Countryman AM Ferreira Filho JBS Ferreira F. Economic assessment of foot-and-mouth disease outbreaks in Brazil. Q Open. (2022) 2:qoac028. doi: 10.1093/qopen/qoac028

[27] De Menezes TC Countryman A Hagerman A Miranda SHG. Economy-wide effects of bovine spongiform encephalopathy in Brazil. J Agric Resour Econ. (2024) 3:489–513. doi: 10.22004/ag.econ.342181

[28] Countryman A Marsh T de Menezes TC Pendell D Rushton J. Economic effects of livestock disease burden in Ethiopia: a computable general equilibrium analysis. Plos One. (2024) 12:e0310268. doi: 10.1371/journal.pone.0310268, 39739717 PMC11687651

[29] Aguiar A Chepeliev M Corong EL van der Mensbrugghe D. The global trade analysis project (GTAP) Data Base: version 11. J Glob Econ Anal. (2022) 7:1–37. doi: 10.21642/JGEA.070201AF

[30] Beckman J Baquedano F Countryman A. The impacts of COVID-19 on GDP, food prices, and food security. Q Open. (2021) 1:qoab005. doi: 10.1093/qopen/qoab005

[31] Hertel TW Tyner WE Birur DK. The global impacts of biofuel mandates. Energy J. (2010) 31:75–100. doi: 10.5547/ISSN0195-6574-EJ-Vol31-No1-4

[32] Sykes AL Galvis JA O’hara KC Corzo C Machado G. Estimating the effectiveness of control actions on African swine fever transmission in commercial swine populations in the United States. Prev Vet Med. (2023) 217:105962. doi: 10.1016/j.prevetmed.2023.105962, 37354739

[33] Frezal C. Gay S.H. Nenert C.. (2021). The impact of the African swine fever outbreak in China on global agricultural markets. OECD food, agriculture, and fisheries. Paper n. 156. Available online at: https://www.oecd.org/content/dam/oecd/en/publications/reports/2021/05/the-impact-of-the-african-swine-fever-outbreak-in-china-on-global-agricultural-markets_ae83db3d/96d0410d-en.pdf (Accessed January 27, 2025).

[34] Chenais E Ahlberg V Andersson K Banihashem F Björk L Cedersmyg M . First outbreak of African swine fever in Sweden: local epidemiology, surveillance, and eradication strategies. Transbound Emerg Dis. (2024) 1:6071781. doi: 10.1155/2024/6071781, 40303069 PMC12017073

[35] Dhal J. Risk assessments and risk mitigation to prevent the introduction of African swine fever into the Danish pig population. Animals. (2024) 17:2491. doi: 10.3390/ani14172491, 39272276 PMC11394020

[36] Driver A.. (2023). Sweden faces pork export bans after ASF discovery. Pigworld, Sep. 12, 2023. Available online at https://www.pig-world.co.uk/news/sweden-faces-pork-export-bans-after-asf-discovery.html (Accessed January 27, 2025].

[37] Beltrán-Alcrudo D Falco JR Raizman E Dietze K. Transboundary spread of pig diseases: the role of international trade and travel. BMC Vet Res. (2019) 64,1–14. doi: 10.1186/s12917-019-1800-5 PMC6387505 30795759

[38] MacLachlan MJ Boussios D Hagerman AD. Market responses to export restrictions from highly pathogenic avian influenza outbreaks. J Agric Resour Econ. (2021) 47:209–24. doi: 10.22004/ag.econ.310527

[39] De Menezes TC Ferreira Filho JBS Countryman A. Potential economic impacts of foot-and-mouth disease in Brazil: a case study for Mato Grosso and Paraná. J Agric Appl Econ Assoc. (2023) 3:481–96. doi: 10.1002/jaa2.73

[40] Lwin WY Schaefer KA Hagerman AD. Animal disease outbreaks and upstream soybean trade. Food Policy. (2024) 127:102685. doi: 10.1016/j.foodpol.2024.102685

[41] Swayne DE Hill RE Clifford J. Safe application of regionalization for trade in poultry and poultry products during highly pathogenic avian influenza outbreaks in the USA. Avian Pathol. (2017) 46:125–30. doi: 10.1080/03079457.2016.1257775, 27817200

[42] Seitzinger A.H. Paarlberg P.L. (2016). Regionalization of the 2014 and 2015 highly pathogenic avian influenza outbreaks. Choices. Quarter 2. Available online at: http://www.choicesmagazine.org/choices-magazine/theme-articles/economic-consequences-of-highly-pathogenic-avian-influenza/regionalization-of-the-2014-and-2015-highly-pathogenic-avian-influenza-outbreaks (Accessed January 30, 2025).

[43] USDA. U.S. Department of Agriculture. (2018). USDA announces regionalization agreement with South Korea to help protect U.S. trade during HPAI detections. Available online at: https://www.usda.gov/about-usda/news/press-releases/2018/03/15/usda-announces-regionalization-agreement-south-korea-help-protect-us-trade-during-hpai-detections (Accessed January 30, 2025).

[44] USDA-FAS. U.S. Department of Agriculture, Foreign Agricultural Service. (2022). Philippines: Highly pathogenic avian influenza import suspensions. Available online at: https://www.fas.usda.gov/data/philippines-highly-pathogenic-avian-influenza-import-suspensions (Accessed January 30, 2025).

[45] UK Department of Environment, Food, & Rural Affairs. (2024). U.S. HPAI regionalization. Imports and EU policy team, OVS NOTE 2024/45. Available online at: http://apha.defra.gov.uk/documents/bip/ovs-notes/2024-45.pdf (Accessed January 30, 2025).

[46] USDA-FAS. U.S. Department of Agriculture, Foreign Agricultural Service. (2024). Advancing trade for U.S. poultry and eggs. Available online at: https://www.fas.usda.gov/sites/default/files/2024-10/US_poultry_eggs_fact_sheet_0.pdf (Accessed January 30, 2025).

[47] British Embassy Manila. (2024). UK poultry imports return to the Philippines. GOV.UK, September 18, 2024. Available online at: https://www.gov.uk/government/news/uk-poultry-imports-return-to-the-philippines (Accessed January 30, 2025).

[48] Corredor D.. (2024). China grants regional authorization to France to import poultry. AviNews, May 15, 2024. Available online at: https://avinews.com/en/china-grants-regional-authorization-to-france-to-import-poultry/ (Accessed January 30, 2025).

[49] Ito S Kawaguchi N Bosch J Aguila-Vega C Sánchez-Vizcaíno JM. What can we learn from the five-year African swine fever epidemic in Asia? Front Vet Sci. (2023) 10:1273417. doi: 10.3389/fvets.2023.1273417, 37841468 PMC10569053

[50] Ma J Chen H Gao X Xiao J Wang H. African swine fever emerging in China: distribution characteristics and high-risk areas. Prev Vet Med. (2020) 175:104861. doi: 10.1016/j.prevetmed.2019.104861, 31810030

[51] Paarlberg PL Seitzinger AH Lee JG Mathews KH. Supply reductions, export restrictions, and expectations for hog returns in a potential classical swine fever outbreak in the United States. J Swine Health Prod. (2009) 17:155–62. doi: 10.54846/jshap/591

[52] Acosta A Lloyd T McCorriston S Lan H. The ripple effect of animal disease outbreaks on food systems: the case of African swine fever on the Chinese pork market. Prev Vet Med. (2023) 215:105912. doi: 10.1016/j.prevetmed.2023.105912, 37119649

[53] Han M Yu W Clora F. Boom and bust in China’s pig sector during 2018-2021: recent recovery from the ASF shocks and longer-term sustainability considerations. Sustainability. (2022) 14:6784. doi: 10.3390/su14116784

[54] Xiong T Zhang W Chen CT. A fortune from misfortune: evidence from hog firms’ stock price responses to China’s African swine fever outbreaks. Food Policy. (2021) 105:102150. doi: 10.1016/j.foodpol.2021.102150

[55] Reuters. (2025). Germany sees meat exports within EU continuing after foot-and-mouth case. Reuters, January 15, 2025. Available online at: https://www.reuters.com/world/europe/germany-sees-meat-exports-eu-continuing-after-foot-and-mouth-case-2025-01-15 (Accessed January 27, 2025).

[56] WTO. World Trade Organization. (2023). European Union advocates for regionalization in trade during animal disease outbreaks. Trade concerns portal. Available online at: https://tradeconcerns.wto.org/en/stcs/details?domainId=SPS&imsId=392 (Accessed January 22, 2025).

[57] United Nations Statistics Division. (2025). UN Comtrade [data set]. International merchandise trade statistics, United Nations statistics division, New York, USA. Available online at: https://comtrade.un.org/data/ (Accessed January 27, 2025).

[58] Domingues EP Haddad EA. Sensitivity analysis in computable general equilibrium models: an application for the regional effects of the free trade area of the Americas (FTAA). Braz Rev Economet. (2005) 1:115–37. doi: 10.12660/bre.v25n12005.2674

[59] Hertel T Hummels D Ivanic M Keeney R. How confident can we be of CGE-based assessment of free trade agreements? Econ Model. (2007) 24:611–35. doi: 10.1016/j.econmod.2006.12.002

