# Supplementary material for: Potential economy-wide impacts of an African swine fever outbreak in the United States
Source: Front Vet Sci. 2026 Feb 2;13:1752899. doi: 10.3389/fvets.2026.1752899 (PMC12908596; doi:10.3389/fvets.2026.1752899)
Supplement: Supplementary file 1 [file Data_Sheet_1.DOCX]

**Supplementary Material**


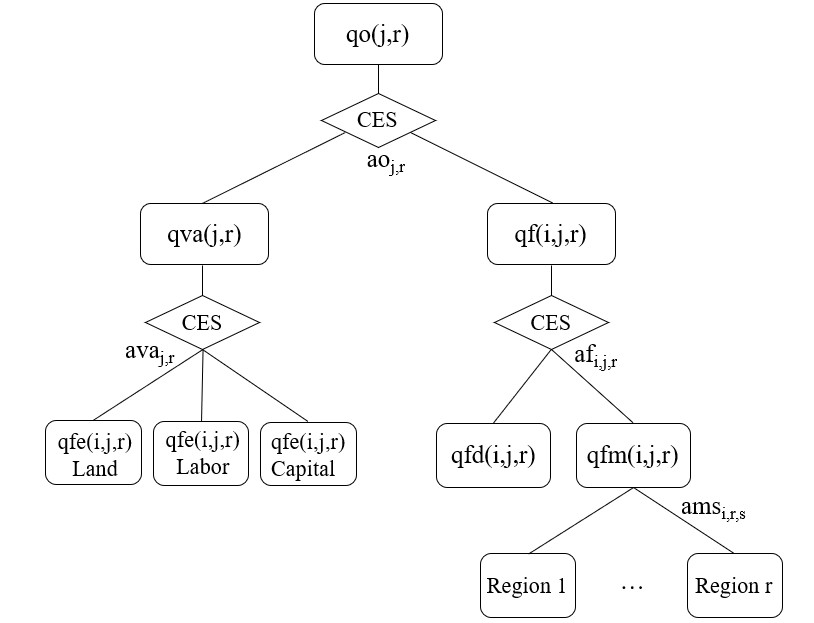


**Figure A.1** Production structure in GTAP version 6.2a

Source: Authors’ design based on GTAP model version 6.2a.

Note: Variable $qo(j,r)$ represents the industry output of commodity $i$ in region $r$; ${ao}_{j,r}$ is the output augmenting technical change in sector $j$ of region $r$; $qva(j,r)$ is the value added in industry $j$of region r; ${ava}_{j,r}$ is the value added augmenting technical change in sector $i$ of region $r$; $qf(i,j,r)$ is the demand for commodity $i$ for use by industry $j$ in region $r$; ${af}_{i,j,r}$ is the composite intermediate input $i$ augmenting technical change by industry j of region $r$; $qfe(i,j,r)$ is the demand for endowment $i$ for use in industry $j$ in region $r$; ${afe}_{i,j,r}$ is the primary factor $i$ augmenting technical change by industry $j$ of region $r$; $qfd(i,j,r)$ is the domestic good $i$ demanded by industry $j$ in region r; $qfm(i,j,r)$ is the quantity of imported tradable $i$ demanded by industry $j$ in region $r$; ${ams}_{i,r,s}$ is the import $i$ from region $r$ augmenting technical change in region $s$; and CES stands for constant elasticity of substitution, which is the production function specification used in the GTAP model.

**Table A.1** Percentage Changes in Hog Bilateral Exports: SO1


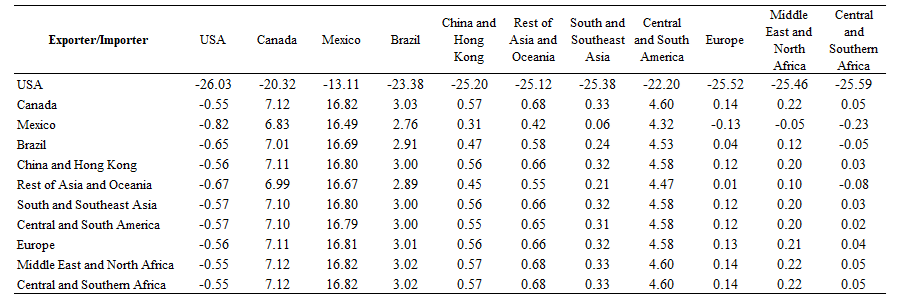


Source: Authors’ simulations

Note: Scenario SO1 considers a hog and pork production decrease of 0.044% and an export loss of 20%.

**Table A.2** Percentage Changes in Hog Bilateral Exports: SO2


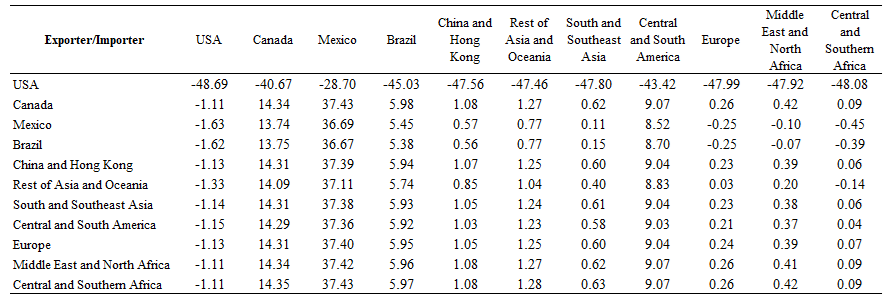


Source: Authors’ simulations

Note Scenario SO2 assumes a hog and pork production decrease of 0.044% and an export loss of 40%.

**Table A.3** Percentage Changes in Hog Bilateral Exports: LO1


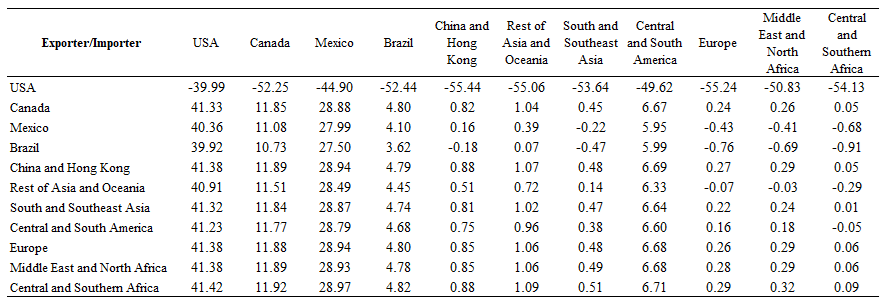


Source: Authors’ simulations

Note: LO1 combines a hog and pork production decrease of 7.33% with an export loss of 40%.

**Table A.4** Percentage Changes in Hog Bilateral Exports: LO2


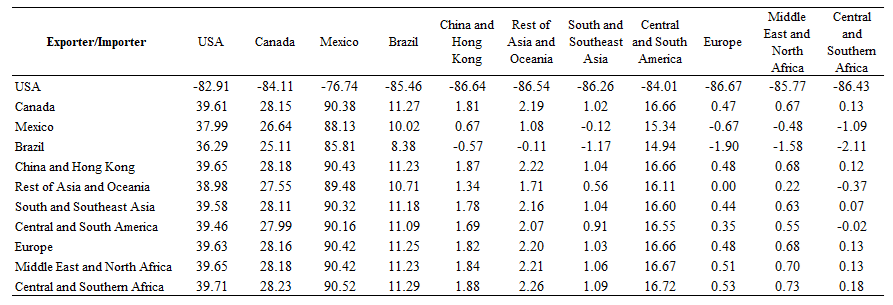


Source: Authors’ simulations

Note: Scenario LO2 represents the worst-case scenario, with a decrease in hog and pork production of 7.33% and an export loss of 80%.

**Table A.5** Percentage Changes in Pork Bilateral Exports: SO1


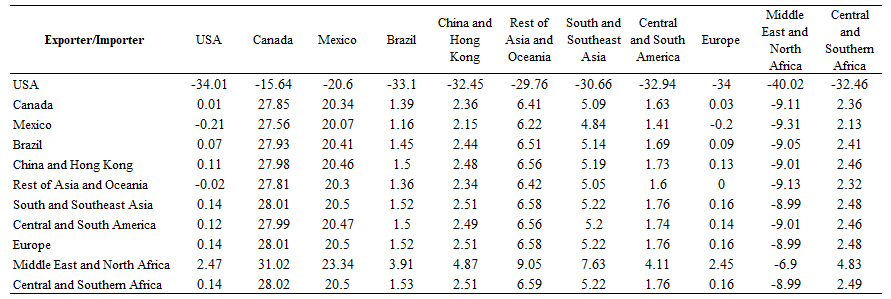


Source: Authors’ simulations

Note: Scenario SO1 considers a hog and pork production decrease of 0.044% and an export loss of 20%.

**Table A.6** Percentage Changes in Pork Bilateral Exports: SO2


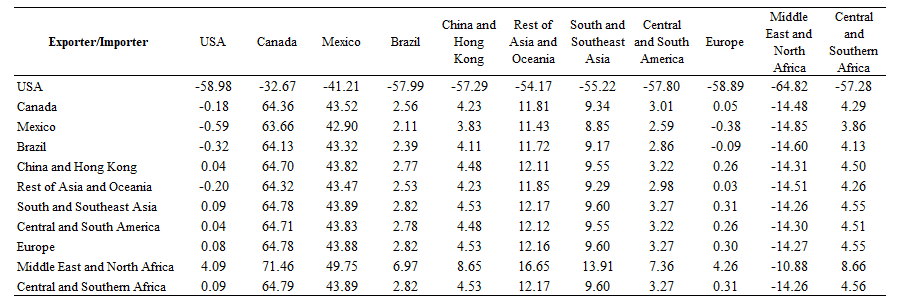


Source: Authors’ simulations

Note: Scenario SO2 assumes a hog and pork production decrease of 0.044% and an export loss of 40%.

**Table A.7** Percentage Changes in Pork Bilateral Exports: LO1


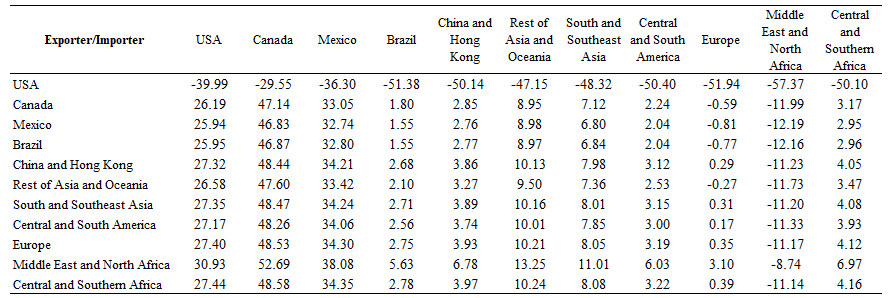


Source: Authors’ simulations

Note: Scenario LO1 combines a hog and pork production decrease of 7.33% with an export loss of 40%.

**Table A.8** Percentage Changes in Pork Bilateral Exports: LO2


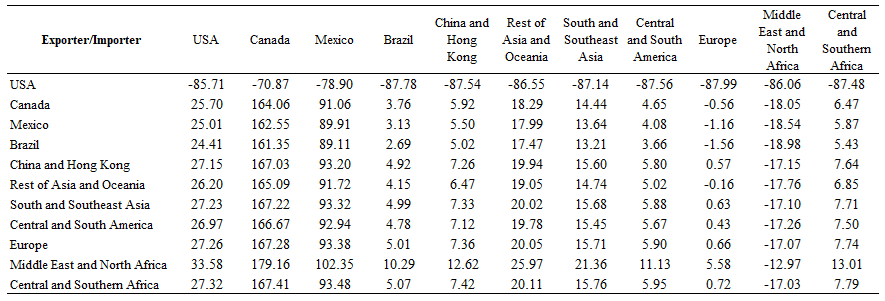


Source: Authors’ simulations

Note: Scenario LO2 represents the worst-case scenario, with a decrease in hog and pork production of 7.33% and an export loss of 80%.

**Table A.9** Decomposition of Changes in Welfare (Equivalent Variation, $US Million): SO1

| **Country** | **Aggregate Welfare Effect** | **Allocative Efficiency Contribution** | **Technological Change Contribution** | **Terms of Trade Contribution** | **Savings and Investment Contribution** |
| --- | --- | --- | --- | --- | --- |
| USA | -310.0 | 45.2 | -37.9 | -257.0 | -59.8 |
| Canada | 45.1 | 8.7 | -64.7 | 63.7 | 37.4 |
| Mexico | 24.1 | 21.4 | -57.3 | 64.2 | -4.1 |
| Brazil | 10.3 | -1.9 | 0.5 | 5.4 | 6.4 |
| China and Hong Kong | -31.1 | -20.6 | -30.7 | 12.4 | 7.9 |
| Rest of Asia and Oceania | -108.0 | -53.6 | -78.0 | 22.0 | 1.4 |
| South and Southeast Asia | -18.0 | -12.2 | -17.5 | 10.1 | 1.6 |
| Central and South America | 1.7 | -2.4 | -5.8 | 10.5 | -0.6 |
| Europe | 62.5 | -2.8 | -7.2 | 64.2 | 8.2 |
| Middle East and North Africa | 44.8 | 18.1 | 23.6 | 2.1 | 1.2 |
| Central and Southern Africa | -9.7 | -6.0 | -7.2 | 2.9 | 0.5 |

Source: Authors’ simulations

Note: Scenario SO1 considers a hog and pork production decrease of 0.044% and an export loss of 20%.

**Table A.10** Decomposition of Changes in Welfare (Equivalent Variation, $US Million): SO2

| **Country** | **Aggregate Welfare Effect** | **Allocative Efficiency Contribution** | **Technological Change Contribution** | **Terms of Trade Contribution** | **Savings and Investment Contribution** |
| --- | --- | --- | --- | --- | --- |
| USA | -563.0 | 87.5 | -37.6 | -499.0 | -114.0 |
| Canada | 90.5 | 17.8 | -126.0 | 125.0 | 74.0 |
| Mexico | 55.9 | 48.2 | -109.0 | 124.0 | -7.9 |
| Brazil | 31.1 | -3.7 | 0.8 | 33.6 | 0.3 |
| China and Hong Kong | -54.9 | -38.7 | -54.7 | 19.3 | 19.3 |
| Rest of Asia and Oceania | -191.0 | -95.8 | -140.0 | 39.7 | 5.0 |
| South and Southeast Asia | -32.9 | -23.4 | -31.2 | 17.8 | 3.9 |
| Central and South America | 2.1 | -4.5 | -10.4 | 18.2 | -1.2 |
| Europe | 109.0 | -11.8 | -12.5 | 117.0 | 16.1 |
| Middle East and North Africa | 69.9 | 28.3 | 39.7 | -0.9 | 2.8 |
| Central and Southern Africa | -18.6 | -11.3 | -12.6 | 4.2 | 1.1 |

Source: Authors’ simulations

Note: Scenario SO2 assumes a hog and pork production decrease of 0.044% and an export loss of 40%.

**Table A.11** Decomposition of Changes in Welfare (Equivalent Variation, $US Million): LO1

| **Country** | **Aggregate Welfare Effect** | **Allocative Efficiency Contribution** | **Technological Change Contribution** | **Terms of Trade Contribution** | **Savings and Investment Contribution** |
| --- | --- | --- | --- | --- | --- |
| USA | -10,905.0 | -66.9 | -10,030.0 | -492.0 | -315.0 |
| Canada | 320.0 | 19.8 | -27.1 | 145.0 | 182.0 |
| Mexico | 120.0 | 58.7 | -24.0 | 92.4 | -7.1 |
| Brazil | 154.0 | -36.6 | 0.1 | 207.0 | -16.6 |
| China and Hong Kong | -72.0 | -88.4 | -10.7 | -43.9 | 71.1 |
| Rest of Asia and Oceania | -349.0 | -249.0 | -33.3 | -110.0 | 44.0 |
| South and Southeast Asia | -77.1 | -70.6 | -6.8 | -16.1 | 16.2 |
| Central and South America | 61.7 | 4.8 | -1.7 | 63.0 | -4.4 |
| Europe | 141.0 | -74.6 | -2.8 | 202.0 | 16.8 |
| Middle East and North Africa | -3.6 | 17.8 | 10.3 | -42.1 | 10.3 |
| Central and Southern Africa | -21.8 | -16.8 | -3.1 | -4.5 | 2.6 |

Source: Authors’ simulations

Note: Scenario LO1 combines a hog and pork production decrease of 7.33% with an export loss of 40%.

**Table A.12** Decomposition of Changes in Welfare (Equivalent Variation, $US Million): LO2

| **Country** | **Aggregate Welfare Effect** | **Allocative Efficiency Contribution** | **Technological Change Contribution** | **Terms of Trade Contribution** | **Savings and Investment Contribution** |
| --- | --- | --- | --- | --- | --- |
| USA | -11,413.0 | 17.6 | -9,937.0 | -1,076.0 | -418.0 |
| Canada | 423.0 | 42.3 | -178.0 | 295.0 | 264.0 |
| Mexico | 207.0 | 131.0 | -145.0 | 235.0 | -14.7 |
| Brazil | 216.0 | -39.9 | 1.0 | 311.0 | -56.5 |
| China and Hong Kong | -111.0 | -120.0 | -65.2 | -26.6 | 101.0 |
| Rest of Asia and Oceania | -480.0 | -312.0 | -170.0 | -49.6 | 52.7 |
| South and Southeast Asia | -103.0 | -91.5 | -37.8 | 3.8 | 22.7 |
| Central and South America | 61.2 | 1.5 | -12.6 | 78.4 | -6.2 |
| Europe | 221.0 | -102.0 | -14.8 | 302.0 | 36.6 |
| Middle East and North Africa | 21.3 | 26.6 | 45.1 | -65.3 | 14.9 |
| Central and Southern Africa | -38.6 | -26.0 | -15.1 | -1.4 | 4.0 |

Source: Authors’ simulations

Note: Scenario LO2 represents the worst-case scenario, with a decrease in hog and pork production of 7.33% and an export loss of 80%.
